# The winter is coming: seasonal variations in BDNF levels among older adults in a high-latitude region — a preliminary study

**DOI:** 10.3389/fpsyt.2025.1692566

**Published:** 2025-10-14

**Authors:** Matías Castillo-Aguilar, Diego Mabe-Castro, Matías Mabe-Castro, Thiago Teixeira Mendes, Yeny Concha-Cisternas, Eduardo Guzmán-Muñoz, Cristian Núñez-Espinosa

**Affiliations:** ^1^ Centro Asistencial Docente y de Investigación (CADI-UMAG), Punta Arenas, Chile; ^2^ Escuela de Medicina, Universidad de Magallanes (UMAG), Punta Arenas, Chile; ^3^ Departamento de Kinesiología, Universidad de Magallanes (UMAG), Punta Arenas, Chile; ^4^ Federal University of Bahia (UFBA), Salvador, Brazil; ^5^ Escuela de Kinesiología, Facultad de Salud, Universidad Santo Tomás, Talca, Chile; ^6^ Vicerrectoria de Innovación e Investigación, Universidad Arturo Prat, Iquique, Chile; ^7^ Escuela de Pedagogía en Educación Física, Facultad de Educación, Universidad Autónoma de Chile, Talca, Chile

**Keywords:** brain-derived neurotrophic factor, seasonal variation, winter, older adults, cognition, anxiety

## Abstract

**Objective:**

To evaluate seasonal changes in plasma Brain-Derived Neurotrophic Factor (BDNF) from the start to the end of winter and identify moderating factors.

**Methods:**

An observational longitudinal study of 17 community-dwelling older adults (mean age 76.4 ± 5.6 years; 12 women) was conducted with assesments performed at the beginning and at the end of the winter, which included multi-frequency bioelectrical impedance analysis for body composition, standardized physical performance testing, cognitive and anxiety screening, and plasma BDNF quantification by enzyme-linked immunosorbent assay. Data were analyzed using hierarchical Bayesian mixed-effects models with time-by-covariate interaction terms; inference was based on posterior medians and 95% highest-density credible intervals.

**Results:**

Plasma BDNF decreased from the first to the second assessment. This association remained after adjustment for baseline cognitive performance and anxiety. Physical performance was not associated with attenuation of the seasonal decline.

**Conclusions:**

In this small longitudinal cohort, winter was associated with a reduction in plasma BDNF in older adults, independent of baseline cognition and anxiety. These results require replication in larger samples.

## 1 Introduction

The global population is undergoing an unprecedented demographic shift, with the proportion of older adults increasing at a faster rate than any other age group (1). This aging phenomenon presents a complex set of public health challenges, chief among them the preservation of cognitive function and the prevention of age-related neurological decline (2, 3). As individuals age, the brain undergoes a series of structural and functional changes that can lead to impairments in memory, executive function, and overall cognitive vitality (2). This decline is not merely a consequence of chronological aging but is profoundly influenced by a complex interplay of genetic predispositions, lifestyle factors, and environmental exposures (2, 4). Consequently, identifying modifiable factors that can bolster the brain’s resilience against the ravages of time is a paramount goal in geriatric medicine and neuroscience. A central focus of this research has been on the molecular mechanisms that support neuronal health, with a particular emphasis on neurotrophic factors that govern neuronal survival, growth, and plasticity (2, 5).

Among these crucial molecules, Brain-Derived Neurotrophic Factor (BDNF) has emerged as a critical regulator of central nervous system health across the lifespan. BDNF is a small, secreted protein of the neurotrophin family that is highly expressed in brain regions essential for learning and memory, such as the hippocampus and cerebral cortex (6). Its biological functions are extensive and vital; BDNF promotes neurogenesis, enhances synaptogenesis, and modulates synaptic plasticity, the cellular basis for learning, memory and pain modulation (6–9). Reductions in circulating and central BDNF levels have been consistently implicated in the pathophysiology of various neurological and psychiatric conditions, including Alzheimer’s disease, Parkinson’s disease, and major depressive disorder (10–12). In the context of healthy aging, lower BDNF levels are associated with poorer cognitive performance and accelerated brain atrophy, making them a critical biomarker of brain vulnerability (10–12). Conversely, interventions known to promote cognitive health, such as physical exercise and cognitive stimulation, have been shown to upregulate BDNF expression (7, 13, 14), suggesting it may mediate the beneficial effects of these lifestyle factors. Therefore, understanding the factors that regulate BDNF levels in older adults is essential for developing strategies to promote successful brain aging.

While intrinsic factors like genetics and lifestyle choices are well-established modulators of BDNF, the influence of broader environmental rhythms, such as seasonal changes, remains a comparatively underexplored frontier, particularly within geriatric populations in high-latitude regions. Humans, like most organisms, are subject to seasonal cycles that influence physiology and behavior (15). The transition into winter in temperate and polar regions is characterized by reduced ambient light, lower temperatures, and often, corresponding changes in diet, physical activity, social interaction, and sleep patterns (15–17). The underlying biology of seasonality is thought to involve disruptions in circadian rhythms and neurotransmitter systems, including serotonin and melatonin, which are intricately linked with light exposure (18, 19). Given that the pathways regulating mood and neuroplasticity are deeply intertwined, it is biologically plausible that the same environmental pressures that affect mood and lifestyle changes could also impact the expression of neurotrophic factors like BDNF.

Preliminary evidence in animal models links seasonality to circulating BDNF (20). Some studies, mainly in younger samples, report lower BDNF in winter (21). However, the effect is not yet systematically characterized in older adults nor is its clinical magnitude known. Older persons may be especially vulnerable because age-related physiological changes, e.g. reduced sunlight-driven neurotransmitter synthesis and altered neurotrophin regulation (22), and winter-associated social factors, like greater fall risk and less outdoor activity, increase exposure to environmental and behavioral stressors that lower BDNF (13, 23). The relationship is unlikely to be uniform: individual differences in demographic (age, sex), somatic (functional performance, adiposity), cognitive (cognitive reserve), and psychological (anxiety, baseline mental health) factors may moderate or confound seasonal effects. Higher physical function and lower adiposity is generally associated with healthier BDNF profiles and may buffer seasonal decline. Nevertheless, paradoxical patterns may also occur, as individuals with initially high BDNF (and better cognitive performance) could experience greater absolute winter decreases (“further to fall”), while anxiety-linked low BDNF levels may interact with seasonality in unpredictable ways. Given this heterogeneity, rigorous longitudinal studies that model multiple covariates and interactions (rather than simple bivariate correlations) are required to quantify winter-related changes in BDNF and identify vulnerable subgroups.

This study aims to fill a critical gap in our understanding of environmental influences on brain health in later life. We seek to provide a rigorous, quantitative characterization of the changes in plasma BDNF levels across the winter season in a cohort of older adults. The primary objective is to determine the existence, direction, and magnitude of seasonal variation in BDNF from the beginning to the end of winter. Our secondary, and arguably more critical objective, is to explore the moderating effects of key individual characteristics. Specifically, we aim to investigate how age, sex, physical performance, body composition, cognitive function, and anxiety symptomatology influence both baseline BDNF levels and its change throughout the winter.

To address these questions, we employed a longitudinal design, measuring variables at two distinct time points bracketing the winter season. We hypothesize that plasma BDNF levels will significantly decrease over the winter period. Furthermore, we hypothesize that this seasonal decline will be moderated by intrinsic factors, expecting that individuals with lower cognitive function, higher anxiety, and poorer physical health will be more susceptible to the negative impact of winter on BDNF homeostasis.

## 2 Material and methods

### 2.1 Study design

This study employed an observational, correlational, and longitudinal design, with a two-time-point data collection measurements.

Prior to any data acquisition, all participants received a comprehensive explanation of the study’s aims, procedures, and potential outcomes. In adherence to ethical standards and to ensure respect for individual autonomy, informed consent was obtained from each participant.

### 2.2 Setting

This study was conducted at the Centro Asistencial Docente e Investigación (CADI-UMAG), the academic healthcare and research center of the University of Magallanes, located in Punta Arenas, Chile. To minimize the impact of circadian rhythms on physiological measurements, all assessments were conducted in a controlled environment between 9:00 and 11:00 a.m.

The evaluation room was maintained at a consistent temperature of 20°C to ensure participant comfort and standardize testing conditions. Consistent illumination was provided by artificial white lighting to prevent variations in ambient light that could affect visual or cognitive factors during the assessments. Furthermore, all evaluations were carried out in a private and quiet setting to minimize external distractions and enhance the reliability of the collected data.

### 2.3 Participants

Participants were recruited through non-probabilistic convenience sampling from the local community through advertisements and outreach. A total of 17 older adults participated in this study, comprising 12 women and 5 men. Eligibility criteria for participation were: (i) being 60 years or older at the time of enrollment; (ii) permanent residency in the Magallanes and Chilean Antarctic region, ensuring a relatively homogeneous population exposed to similar environmental and socioeconomic factors; (iii) achieving a score above 60% on the Karnofsky Performance Status scale, a common measure of functional capacity, which indicates sufficient autonomy to complete the study assessments (24); and (iv) no prior diagnosis of conditions that could confound autonomic or cardiovascular function, such as diabetic neuropathy, pacemaker implantation, clinical depression, cognitive impairment, motor disability, or dementia.

### 2.4 Procedures

Participants attended a single study visit after a 12-hour overnight fast and abstention from strenuous exercise and alcohol. On arrival they provided informed consent and underwent a brief health screening (including verification of inclusion/exclusion criteria and resting blood pressure: systolic<140 mmHg and diastolic<90 mmHg). Sociodemographic and medical history data were obtained by structured interview.

Body composition was measured first using the Tanita BC-558 multi-frequency bioelectrical impedance analysis (BIA) following device and manufacturer recommendations (participants had fasted ≥4 h, emptied their bladder, stood barefoot and held hand electrodes). Immediately after BIA and prior to any physical testing, a peripheral venous blood sample was drawn into EDTA tubes by a trained nurse. Tubes were gently inverted, centrifuged (1,500 × g, 10min, 4°C), plasma aliquoted and stored at −80°C; all processing occurred within two hours of collection to preserve BDNF integrity.

After a minimum 10-minute seated rest, the Short Physical Performance Battery (SPPB) was administered by a trained kinesiologist (balance, 4-m gait speed and five repeated chair rises; scored 0–12). Blood pressure was assessed immediately after the SPPB for safety monitoring. Cognitive status (Montreal Cognitive Assessment, MoCA, validated Spanish version) and anxiety symptoms (Beck Anxiety Inventory, BAI, Spanish version) were then administered in a quiet room by trained personnel: MoCA as a structured interview and BAI as a self-report questionnaire (assistance provided when necessary). All instruments were applied according to standardized, published protocols.

### 2.5 Assessments

#### 2.5.1 Body composition

Body composition, including body mass index (BMI), body fat percentage, lean muscle mass, body water, and bone mass, was assessed using multi-frequency BIA. Measurements were performed using the Tanita BC-558 Ironman Segmental Body Composition Monitor (Tanita Ironman, Arlington Heights, IL 60005 USA). Participants were instructed to fast for at least 4 hours prior to the assessment and to avoid strenuous exercise and alcohol consumption for at least 12 hours. They were also asked to empty their bladder before the measurement. Participants stood barefoot on the device’s foot electrodes and held the hand electrodes while wearing light clothing.

#### 2.5.2 Short physical performance battery

The Short Physical Performance Battery (SPPB) consisted of three standardized components: balance, gait speed, and lower-limb strength (25). Balance testing included side-by-side, semi-tandem, and tandem positions held for up to 10 seconds each. Gait speed was measured over a 4-meter straight course at usual pace; two trials were performed and the faster time was recorded. Lower-limb strength was assessed by timing five repeated unassisted chair rises from a straight-backed chair (seat height ~43 cm). Each task was scored per established criteria (0–4 points) and summed to a 0–12 composite score. A trained kinesiologist administered the tests and ensured participant safety.

#### 2.5.3 Brain derived neurotrophic factor

BDNF was quantified in the peripheral blood of each participant using a sandwich immunoassay (ELISA) (RayBio^®^ Human BDNF ELISA, cat. ELH-BDNF, detection range: 80 pg/ml - 16 ng/ml, sensitivity: 80 pg/ml, based on manufacturer documentation). Venipunctures were performed in the morning on a fasted state, and patients were advised to avoid vigorous exercise, caffeine, and alcohol for the previous 24h. Plasma samples were obtained; after centrifugation, the supernatant was aliquoted and stored until analysis. The assay uses 96-well microplates coated with anti-BDNF antibody; the analyte is captured and detected with biotinylated antibody, followed by streptavidin-conjugated hydrogen peroxidase (HRP) and chromogenic development with TMB; the reaction is stopped with sulfuric acid, and the absorbance is recorded at 450 nm by an absorbance microplate reader (Tecan Trading AG, Switzerland). The standard curve was prepared by reconstituting the BDNF standard and performing serial dilutions in Assay Diluent A (serum/plasma matrix) to obtain a series of concentrations between 0.066 and 16 ng/mL, together with the blank (0 ng/mL). The samples were diluted in Assay Diluent A within the recommended range for serum/plasma (1:50), according to the expected concentration. The analytical procedure was executed according to the manufacturer’s protocol.

#### 2.5.4 Montreal cognitive assessment

Global cognitive functioning was evaluated using the Montreal Cognitive Assessment (MoCA) (26). The MoCA is a 30-point cognitive screening tool designed specifically to be sensitive in detecting mild cognitive impairment. It comprehensively assesses multiple cognitive domains, including short-term memory recall, visuospatial and executive functions, attention, concentration, working memory, language, and orientation to time and place. A total score of 26 or above is generally considered to be within the normal range. Due to its brevity and high sensitivity, the MoCA is a widely accepted instrument for assessing cognitive status in older adult populations in both clinical and research settings (27).

#### 2.5.5 Beck anxiety questionnaire

Anxiety symptomatology was measured with the Beck Anxiety Inventory (BAI) (28). The BAI is a 21-item self-report questionnaire that quantifies the severity of anxiety symptoms experienced over the past week. Each item is rated on a 4-point Likert scale, from 0 (“Not at all”) to 3 (“Severely”), reflecting the degree to which the individual has been bothered by each symptom. The total score ranges from 0 to 63, with higher scores indicating more severe levels of anxiety. The BAI is a robust and reliable instrument valued for its ability to measure the somatic and affective components of anxiety (29).

### 2.6 Statistical analysis

#### 2.6.1 Framework

We employed a fully Bayesian modeling framework to characterize how cardiac autonomic modulation responds to exercise in the presence of multiple confounding influences. Bayesian inference was preferred over traditional frequentist methods because it provides complete posterior distributions for all parameters, thereby allowing thorough quantification of uncertainty and probabilistic interpretation via credible intervals (30).

For descriptive statistics, continuous variables are summarized as mean and standard deviation (M ± SD), while categorical variables are reported using absolute counts (n) and relative frequencies (%).

#### 2.6.2 BDNF model

In order to explore the effect of seasonal changes and the influence of other factors, we employed a Bayesian generalized linear mixed effects model, depicted in Equation 1.


(1) $$\begin{array}{rr} {\text{BDNF}}_{i} & ∼N\left(\mu_{i},\sigma^{2}\right)\\ \mu_{i} & =\beta_{0}+\sum_{k=1}^{K}\beta_{k}X_{ik}+\beta_{\text{time}}{\text{Time}}_{i}+\sum_{k=1}^{K}\beta_{k,\text{time}}\left(X_{ik}\times {\text{Time}}_{i}\right)+\gamma_{j\left[i\right]}\\ \gamma_{j} & ∼N\left(0,\tau^{2}\right),\text{ with }j=1,\ldots ,J \end{array}$$

Where ${\text{BDNF}}_{\text{i}}$ corresponds to the standardized outcome (response) variable for each i observation, normally distributed around $\mu_{i}$ with constant variance $\sigma^{2}$ . The estimated effect $\mu_{i}$ is a linear combination of a global intercept $\beta_{0}$ and the predictor coefficients $\beta_{k}$ for all standardized $X_{ik}$ variables for all k parameters. Additionally, in $\sum_{k=1}^{K}\beta_{k,\text{time}}\left(X_{ik}\times {\text{Time}}_{i}\right)$ we consider a time-interaction term to recover the effects of confounding variables in modifying the temporal trajectories of plasmatic BDNF levels. Finally, $\gamma_{j\left[i\right]}$ denotes the random intercept for subject j distributed as $\gamma_{j}∼N\left(0,\tau^{2}\right)$ , where $\tau^{2}$ is the between-subjects variance of the random intercept.

The prior distributions used were specified as weakly regularizing normal distributions centered around 0 for the standard deviation of random intercepts $\tau ∼N^{+}\left(0,3\right)$ and the linear coefficients for the estimated effects β∼N(0,3) , By incorporating prior information, specified as weakly regularizing distributions, we constrained implausible parameter values, mitigated the impact of outliers, and improved convergence during model fitting.

#### 2.6.3 Parameter estimation

Bayesian estimation employed the No-U-Turn Sampler (a variant of Hamiltonian Monte Carlo) as implemented in the *brms* (v2.22.0) and *rstan* (v2.32.7) packages (31, 32), in the R language (v4.5.0) (33). For each multivariate model, four Markov chains were run, each with 10,000 warm-up iterations followed by another 10,000 sampling iterations, drawing every 8th sample to reduce autocorrelation among chains, resulting in 5,000 post-warmup samples per parameter.

To verify sampling convergence and stability, we checked that the potential scale reduction factor ( $\hat{R}$ ) for every parameter was below 1.01 and that the effective sample size exceeded 1,000. We also inspected trace plots visually to confirm adequate chain mixing and performed posterior predictive checks to ensure the model’s predicted distributions aligned with the observed data. The complete posterior distribution, along with traceplots, can be seen in **Supplementary Figure S1**, and posterior predictive checks in **Supplementary Figure S2** from the **Supplementary Material**.

#### 2.6.4 Statistical reporting

Inference followed the SEXIT (Sequential Effect eXistence and Significance Testing) framework (34). For each estimated parameter and its corresponding posterior distribution, we report the posterior median and its 95% highest-density credible interval (HDI). We also present the probability of direction (pd) as a quantitative measure of effect existence. Practical significance (ps) is indexed by the proportion of the posterior mass falling outside a region of practical equivalence (ROPE). The ROPE was defined as ±0.1 times the standard deviation of the response variable, and to ensure consistency all predictors were standardized before to modeling (34).

## 3 Results

### 3.1 Sample characterization

The final sample size included in the study consisted of 17 individuals (age: 76.4 ± 5.6, from 66 to 86 years old), with 12 female and 5 male individuals. Sample characteristics can be observed in **Table 1**. The average BDNF concentrations at the beggining and end of winter were 0.54 ± 0.40 ng/ml and 0.30 ± 0.24 ng/ml, respectively.

**Table 1:** Descriptive statistics of the overall collected sample, aggregated for sex.

Characteristic	Overall N = 17	Male N = 5	Female N = 12	Difference	95% CI
Age (years old)	76.41 ± 5.59	76.40 ± 5.41	76.42 ± 5.90	0.00	-1.0, 1.0
BMI (km/m²)	31.54 ± 4.73	29.92 ± 1.78	32.22 ± 5.45	-0.60	-1.7, 0.47
Muscle mass (kg)	46.79 ± 9.79	59.76 ± 4.80	41.39 ± 4.78	4.1	2.4, 5.9
Body fat (%)	37.08 ± 9.11	26.36 ± 5.97	41.54 ± 5.82	-2.8	-4.2, -1.4
Plasma BDNF (ng/ml)	0.54 ± 0.40	0.52 ± 0.54	0.54 ± 0.36	-0.06	-1.1, 0.98
SPPB score	9.71 ± 2.08	10.60 ± 0.89	9.33 ± 2.35	0.75	-0.32, 1.8
MoCA score	21.94 ± 3.19	23.20 ± 3.27	21.42 ± 3.15	0.60	-0.46, 1.7
BAI score	11.94 ± 10.48	9.20 ± 10.13	13.08 ± 10.84	-0.40	-1.5, 0.65

Data is presented as mean ± standard deviation. Additionally, the standardized mean difference per sex and 95% confidence interval (CI) is presented for each variable.

### 3.2 Seasonal variations of plasma BDNF levels

**Figure 1:**
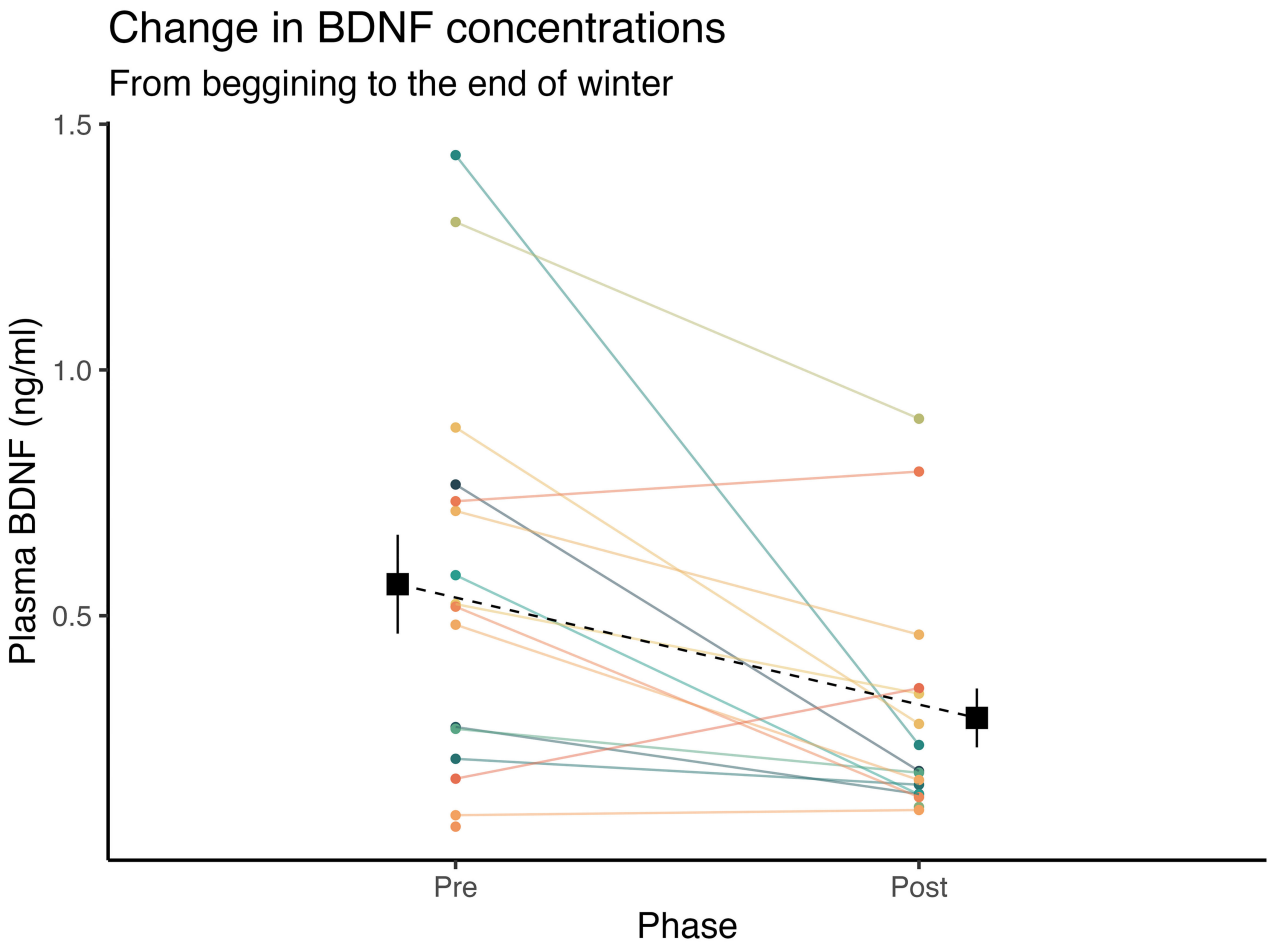
Inter-individual temporal fluctuations in plasmatic BDNF concentrations from the beginning, to the end of winter. Square points and dashed lines represent the mean value, and error bars indicate the standard error of the mean.

When assessing the seasonal effects of winter on plasmatic BDNF levels, we observed a 99.6% posterior probability of a marked decrease in BDNF at the end of winter compared to early seasonal measurements ( β = -0.79, CI_95%_[-1.34, -0.22], pd = 99.6%, ps = 99%). In adjusted models, accounting for the marginal effect of physical and psychological variables, we observed a 71.4% posterior probability of a marked decrease in BDNF at the end of winter ( β = -0.84, CI_95%_[-3.47, 2.07], pd = 71.4%, ps = 69.1%). These effects can be seen in **Figure 1**.

### 3.3 Moderating factors

#### 3.3.1 Age

When inspecting the effect of other moderating variables from fully adjusted models, we observed that greater age was associated with a 79.4% posterior probability of lower BDNF in general ( β = -0.3, CI_95%_[-1.06, 0.42], pd = 79.4%, ps = 70.3%), but age itself did not influence the BDNF trajectories from the beginning to the end of winter ( β = -0.1, CI_95%_[-1.16, 0.89], pd = 57.5%, ps = 50.2%).

#### 3.3.2 Sex

Females, in contrast to males, were linked to a 70.1% posterior probability of having a higher plasma BDNF level ( β = 0.71, CI_95%_[-1.95, 3.59], pd = 70.1%, ps = 67.1%). However, as seen with age, being female was not a protective factor against seasonal-related changes in plasmatic BDNF leves ( β = 0.07, CI_95%_[-3.24, 3.44], pd = 51.5%, ps = 49.3%).

#### 3.3.3 Physical performance (SPPB)

Physical performance was not associated with different values of plasmatic BDNF ( β = -0.18, CI_95%_[-1.31, 0.96], pd = 62.9%, ps = 55.6%), nor with different seasonal trajectories of BDNF to the end of winter ( β = -0.45, CI_95%_[-2.53, 1.58], pd = 68%, ps = 64.2%).

#### 3.3.4 Body composition

In relation to body composition variables, we observed that body fat, not muscle mass nor body mass index, was associated with a 73.4% posterior probability of a lower plasmatic BDNF levels ( β = -0.57, CI_95%_[-2.51, 1.2], pd = 73.4%, ps = 69.8%), but neither body mass index ( β = -0.3, CI_95%_[-2.27, 1.61], pd = 62%, ps = 57.9%), body fat ( β = 0.32, CI_95%_[-1.93, 2.46], pd = 61.7%, ps = 58%), nor muscle mass ( β = 0.47, CI_95%_[-1.74, 2.54], pd = 66.8%, ps = 63.3%), was linked to different winter-associated seasonal trajectories in plasmatic BDNF levels.

#### 3.3.5 Cognitive functioning (MoCA)

**Figure 2:**
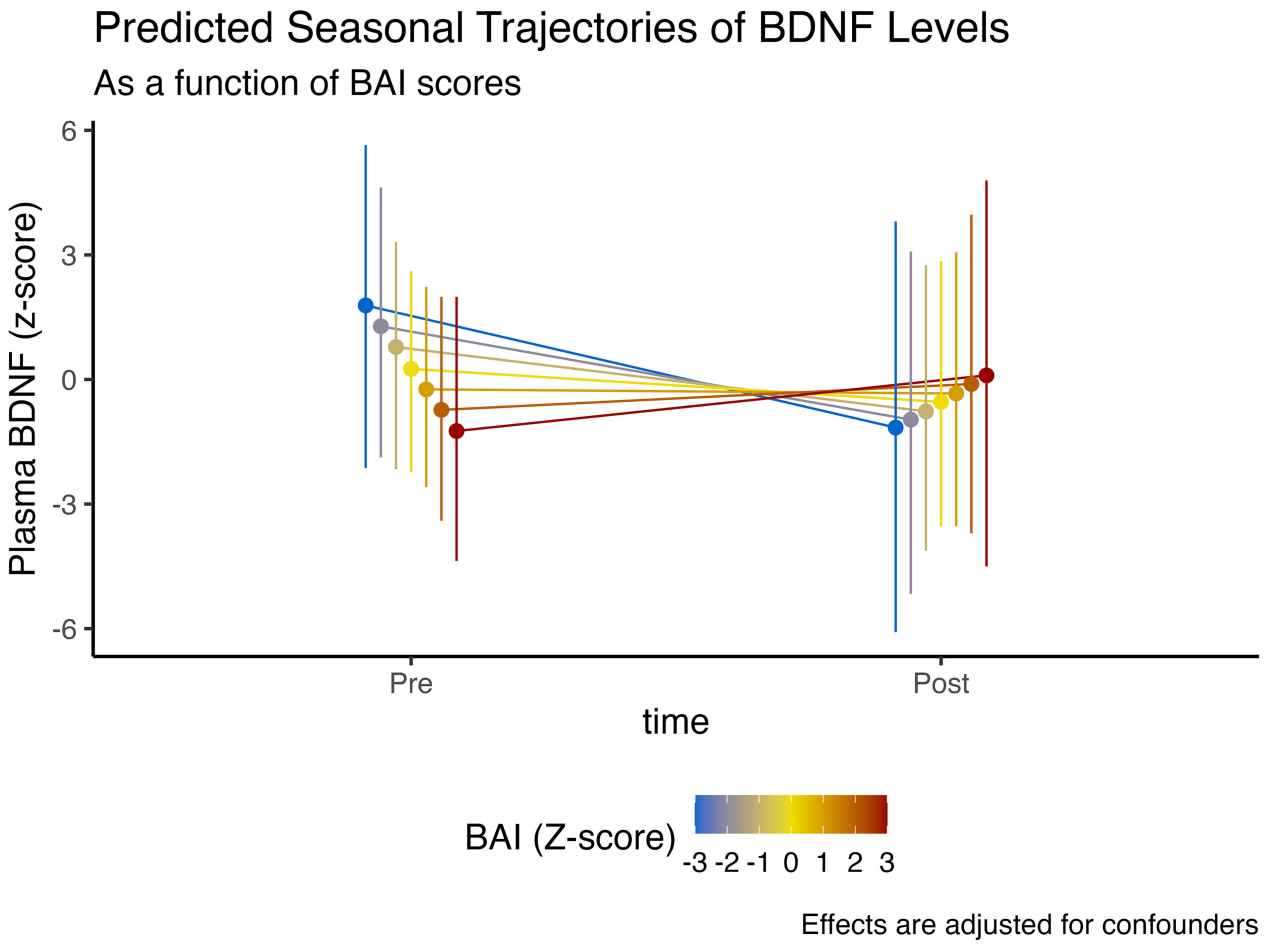
Expected model-predicted and winter-associated seasonal trajectories in plasma BDNF levels, as a function of MoCA performance (z-score). Data is presented as median and 95% highest density interval (HDI). Displayed effects are adjusted for confounding factors and interindividual variability (i.e., random effects structure).

We observed that higher cognitive function, denoted by higher MoCA scores, was associated with an 85.1% posterior probability of higher plasmatic BDNF levels ( β = 0.48, CI_95%_[-0.47, 1.44], pd = 85.1%, ps = 80.1%). However, those with higher baseline cognitive function had a 79.3% posterior probability of experiencing a more abrupt decline in BDNF levels at the end of winter ( β = -0.48, CI_95%_[-1.71, 0.8], pd = 79.3%, ps = 74.5%). These effects are illustrated in **Figure 2**.

#### 3.3.6 Anxiety symptomatology

In regards to anxiety symptomatology, assessed through higher Beck anxiety scores, we observed an 86.6% posterior probability that higher anxiety was linked to lower plasmatic BDNF levels ( β = -0.5, CI_95%_[-1.41, 0.35], pd = 86.6%, ps = 81.6%). However, having more anxiety was associated with a 87.8% posterior probability of more BDNF at the end of winter ( β = 0.72, CI_95%_[-0.48, 1.96], pd = 87.8%, ps = 84.5%).

**Figure 3:**
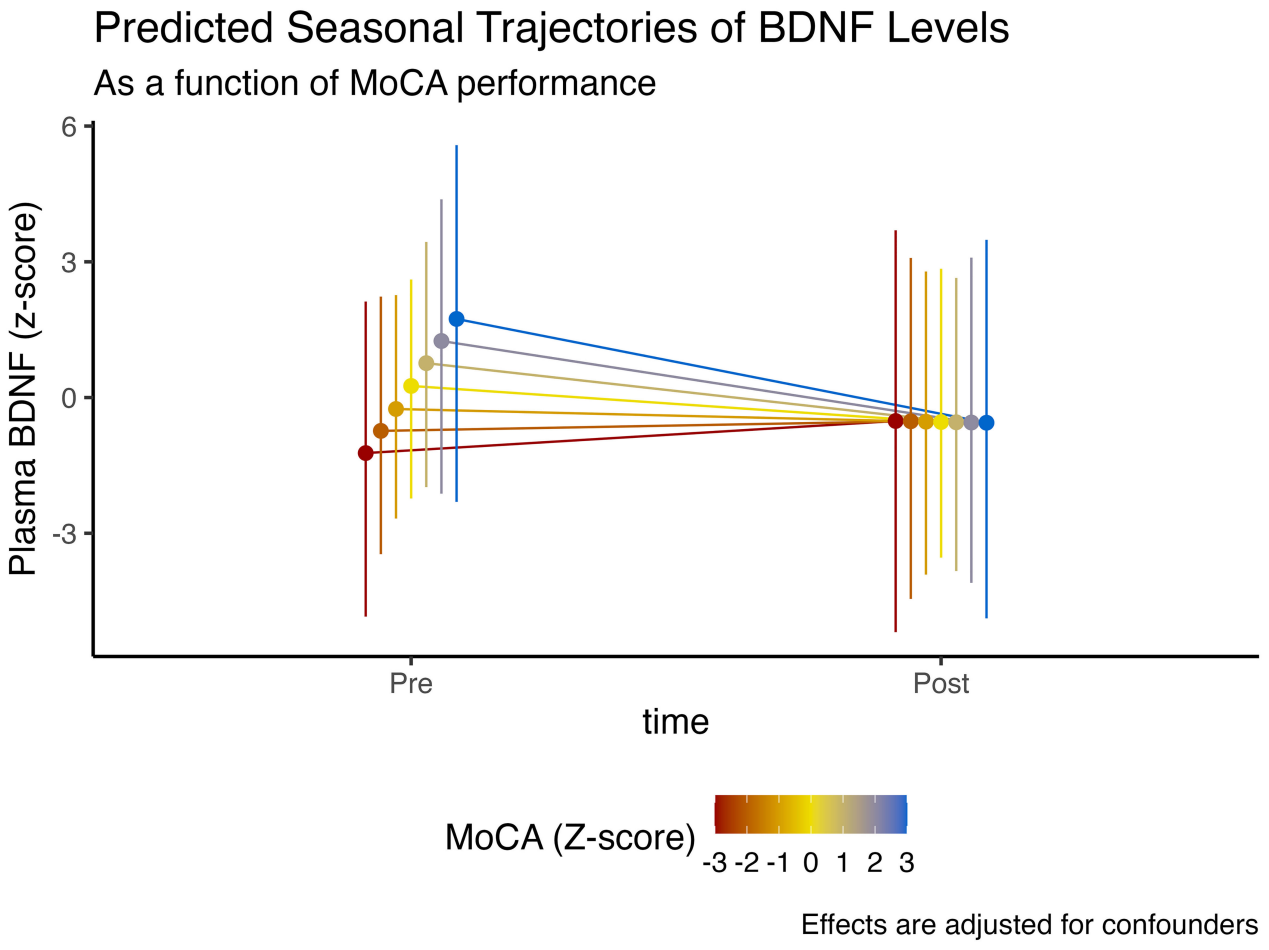
Expected model-predicted and winter-associated seasonal trajectories in plasma BDNF levels, as a function of BAI scores (z-score). Data is presented as median and 95% highest density interval (HDI). Displayed effects are adjusted for confounding factors and interindividual variability (i.e., random effects structure).

These findings, nonetheless, could have an alternative explanation. Individuals who exhibit lower anxiety symptomatology tend to have higher plasmatic BDNF levels at baseline, similarly in individuals with better cognitive function. However, these non-anxious/better-cognition individuals also display the steepest drop in BDNF levels at the end of winter, similar to the observed time interaction effect with MoCA scores. These effects are illustrated in **Figure 3**.

## 4 Discussion

This study investigated the seasonal effects of winter on plasma BDNF levels in a cohort of older adults, employing a robust Bayesian analytical framework to explore the moderating influence of various physiological and psychological factors. Our principal finding reveals a compelling and statistically robust decrease in circulating BDNF from the beginning to the end of the winter season. This primary observation was further nuanced by a series of complex interactions. Notably, we found that individuals with higher baseline cognitive function and lower anxiety symptomatology (i.e., characteristics associated with higher initial BDNF levels) experienced the most pronounced seasonal decline. In contrast, while factors such as advanced age and higher body fat were associated with lower BDNF levels overall, they did not significantly alter the magnitude of the winter-associated drop. These findings collectively suggest that winter represents a period of significant neurobiological challenge for older adults, exerting a potent and convergent effect on a key marker of brain health, and that this vulnerability extends even to individuals who are otherwise cognitively and psychologically robust.

### 4. 1 The effect of the winter season on BDNF

The primary finding of a marked decline in BDNF over the winter months aligns with and extends emerging evidence from other populations (21, 22), providing a crucial piece of the puzzle within the context of geriatric neurobiology. The mechanisms driving this seasonal suppression are likely multifactorial, stemming from a combination of physiological and behavioral changes inherent to the winter season (16, 18, 19). Physiologically, the most cited mechanism is the reduction in photoperiod, or ambient light exposure (35). Light is a powerful synchronizer for the human circadian system, and its reduction during winter is known to disrupt the synthesis of key neurotransmitters, which in turn can influence the expression of BDNF (36). Furthermore, reduced sunlight exposure leads to lower endogenous neurosteroid hormones (like vitamin D) that has been shown to regulate BDNF gene expression in humans (17, 37) and animal models (38). The potent seasonal effect observed in our study lends strong support to the hypothesis that these fundamental biological pathways are significantly impacted during winter.

Behaviorally, winter often imposes a more sedentary and isolated lifestyle on older adults (39). Decreased temperatures, inclement weather, and fear of falling on snow or ice can severely limit outdoor physical activity, which is one of the most powerful known stimuli for BDNF production (13, 14, 40). This reduction in activity is often coupled with diminished social engagement, another factor known to support cognitive and brain health (14, 41). Therefore, the observed decline in BDNF is likely not merely a passive physiological response to reduced light exposure, but rather an active consequence of a seasonal behavioral shift. In this sense, the winter environment creates a “perfect storm” of reduced physiological stimulation and behavioral engagement, culminating in the suppression of crucial neurotrophic support systems. Our findings firmly establish this phenomenon within an older adult cohort, a population uniquely vulnerable to both the causes (e.g., physical frailty) and consequences (e.g., cognitive decline) of reduced BDNF.

### 4.2 The moderating role of cognition and anxiety

Perhaps the most intriguing and clinically relevant finding of this study is the interaction between baseline cognitive and psychological status on seasonal BDNF trajectories. We observed that individuals with higher cognitive scores (MoCA) and lower anxiety, profiles indicative of better brain health and higher baseline BDNF, demonstrated the steepest decline in BDNF over the winter. Conversely, individuals with higher anxiety, who started with lower BDNF, showed a blunted decline. This suggests that being “healthier” at the outset does not confer immunity to seasonal effects; rather, it may be associated with a greater magnitude of seasonal loss.

We interpret this as evidence for a “convergent effect” of winter. This hypothesis posits that winter acts as a powerful, overarching environmental pressure that drives BDNF levels towards a common, lower threshold, irrespective of an individual’s starting point. Those with a higher reserve of BDNF simply have a greater potential distance to fall to reach this winter-induced nadir. This finding challenges the simplistic notion that high cognitive reserve is uniformly protective. While it may provide a buffer against cognitive symptoms, it does not appear to protect against the underlying neurobiological shift in BDNF expression. This has profound implications, suggesting that even the most cognitively robust older adults are susceptible and may warrant targeted preventative strategies during winter. The data points towards winter as a potent equalizer, imposing a neurobiological burden on the aging population as a whole.

### 4.3 Stable influences against seasonal modulators

In contrast to the dynamic interactions observed with cognition and anxiety, our analysis revealed that intrinsic factors, such as age and body composition, acted as stable, non-interactive predictors of BDNF. Specifically, greater age and higher body fat percentage were associated with lower BDNF levels overall, a finding that is well-supported by the broader literature (42, 43). Chronic low-grade inflammation originating from adipose tissue is known to suppress neurotrophic factors, and the aging process itself is characterized by a general decline in anabolic and regenerative processes, including BDNF synthesis (44). This suggests that while age and adiposity may set a lower “baseline” for an individual’s BDNF profile, the seasonal effect of winter is an independent, additive stressor. It is an acute-on-chronic challenge, where the environmental pressures of winter superimpose a further decline on top of pre-existing risk factors, rather than exacerbating their specific influence. Similarly, the lack of a significant moderating effect of physical performance suggests that an individual’s functional capacity may not be as relevant as their actual behavioral change during winter, a variable not directly captured in our study.

### 4.4 Strengths, limitations and future directions

The present study has several notable strengths, including its longitudinal pre-post winter design, the focus on a clinically important and vulnerable geriatric population residing in a high-latitude region, and the application of a sophisticated Bayesian mixed-effects modeling approach. This statistical framework allowed us to move beyond simple p-values to quantify the probability of effects and their uncertainty, providing a more nuanced interpretation of complex interactions (30). However, our findings must be considered in light of several limitations. The most significant is the modest sample size (N=17), which, while sufficient to detect the main effect of season, necessitates caution in interpreting the more complex interaction effects. The wide credible intervals for some parameters reflect this uncertainty, and our results should be considered preliminary until replicated in larger, more diverse cohorts. Additionally, the non-probabilistic sampling, while convenient in observational and exploratory designs, inevitably undermines the generalizability of our findings.

Furthermore, our study relied on plasma BDNF as a peripheral proxy for central BDNF levels. While an association is established, it is an indirect measure of the neurobiological processes occurring within the brain. Lastly, we did not directly measure the proposed mechanistic factors, such as daily light exposure, physical activity levels (e.g., via accelerometry), or Vitamin D status. Future research should aim to address these limitations by incorporating larger sample sizes and employing a multi-modal approach that includes wearable sensor technology and parallel measurement of related biomarkers. Such studies could confirm the “convergent effect” hypothesis and elucidate the precise contributions of light, activity, and diet to the seasonal BDNF decline.

### 4.5 Clinical implications

Despite its limitations, our study provides a clear and compelling message: the winter is coming, and with it, a tangible neurobiological challenge for older adults. The findings suggest that winter is a period of heightened risk, where a key factor for neuronal resilience is significantly diminished across the board. This has direct clinical implications, highlighting the potential for targeted, seasonal interventions to support healthy brain aging. Strategies that could include proactive implementation of structured indoor exercise programs to compensate for seasonal changes in sedentary behavior. The observation that even cognitively healthy individuals experience a sharp decline suggests that such interventions should be considered for the broader older adult population, not just those with pre-existing cognitive deficits.

## 5 Conclusion

Our study shows a marked decline in plasma BDNF levels over the winter season in older adults, including those with high baseline cognitive function. These findings suggest that seasonal variations should be considered as a relevant environmental influence on brain health in aging, especially in high latitude regions. Recognizing this potential vulnerability may inform the design of future preventive strategies aimed at preserving cognitive vitality during winter months.

## Data Availability

The original contributions presented in the study are included in the article/**Supplementary Material**. Further inquiries can be directed to the corresponding author.
