# Supplementary figures and images for: The winter is coming: seasonal variations in BDNF levels among older adults in a high-latitude region — a preliminary study
Source: Front Psychiatry. 2025 Oct 14;16:1692566. doi: 10.3389/fpsyt.2025.1692566 (PMC12560003; doi:10.3389/fpsyt.2025.1692566)

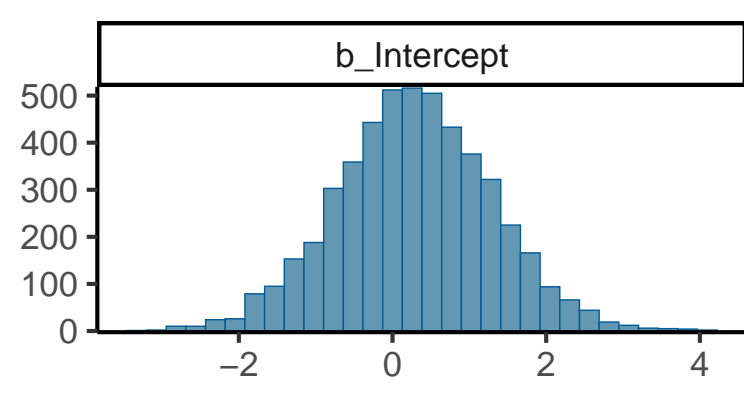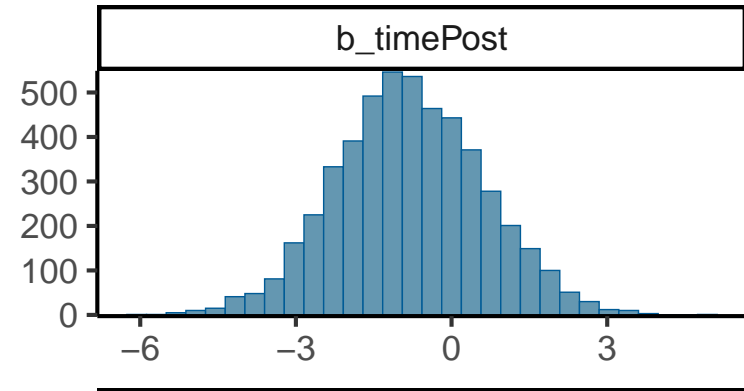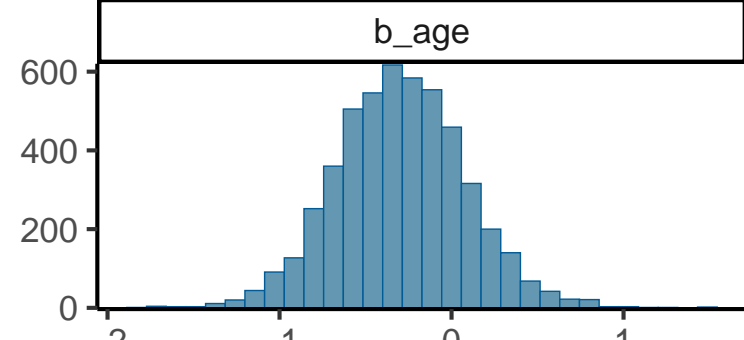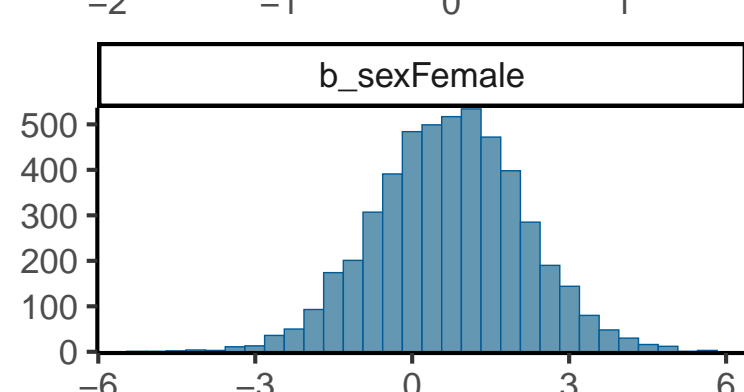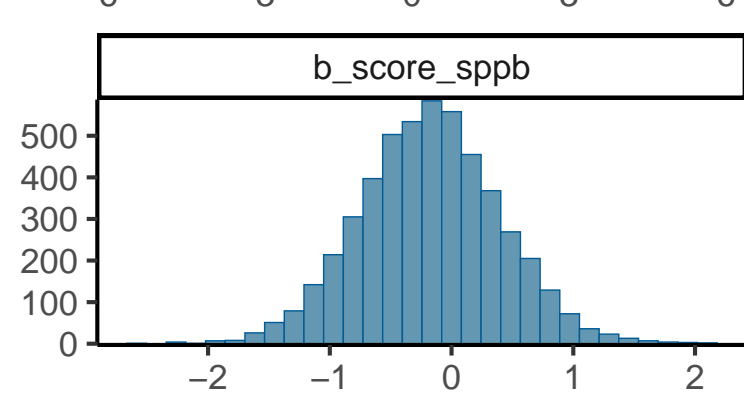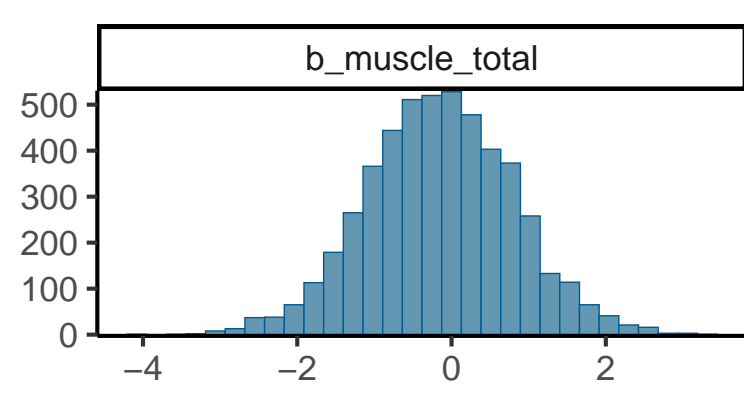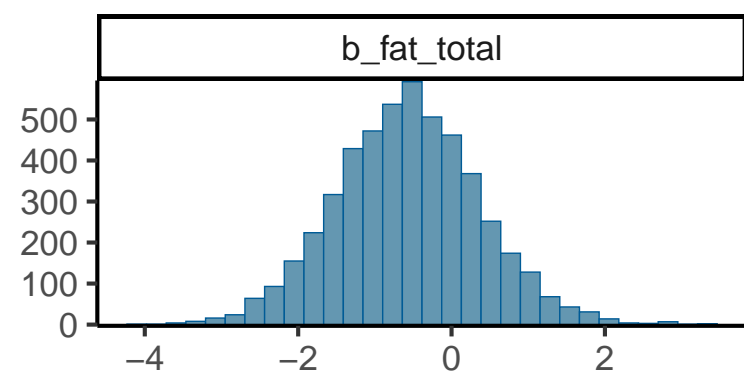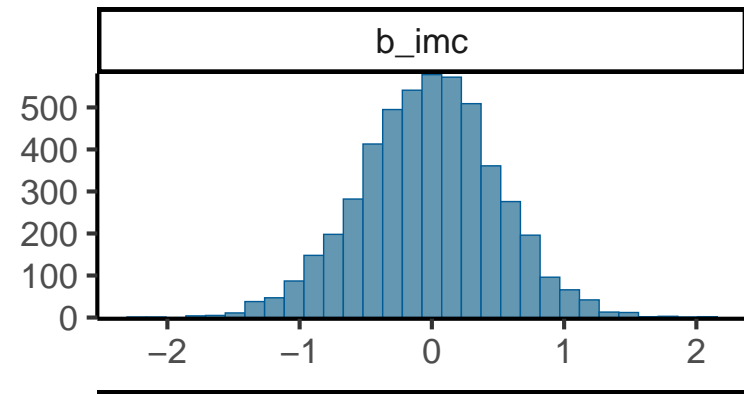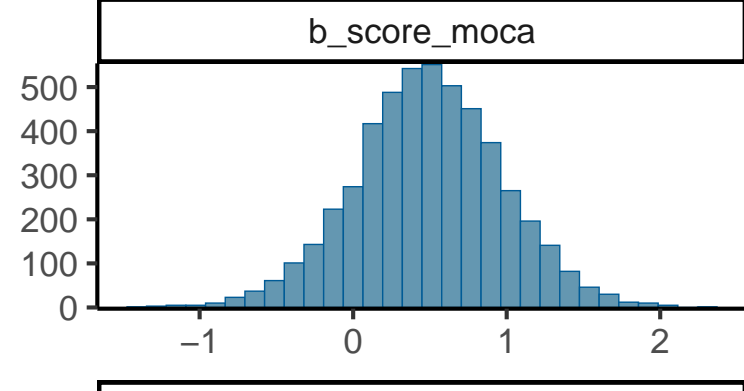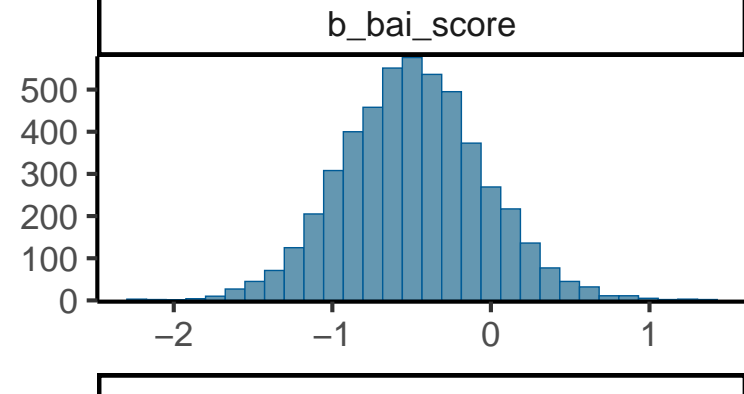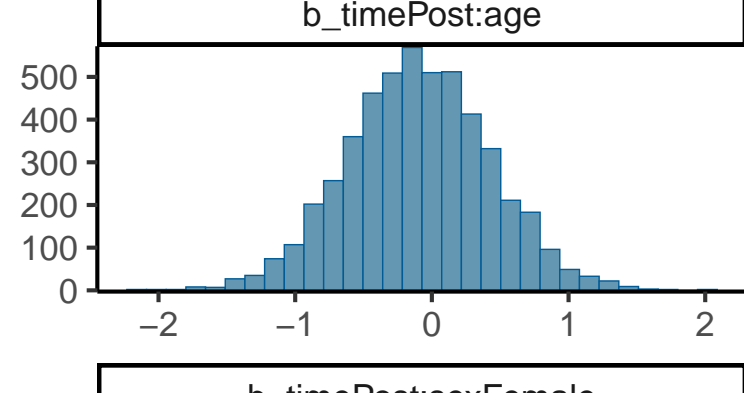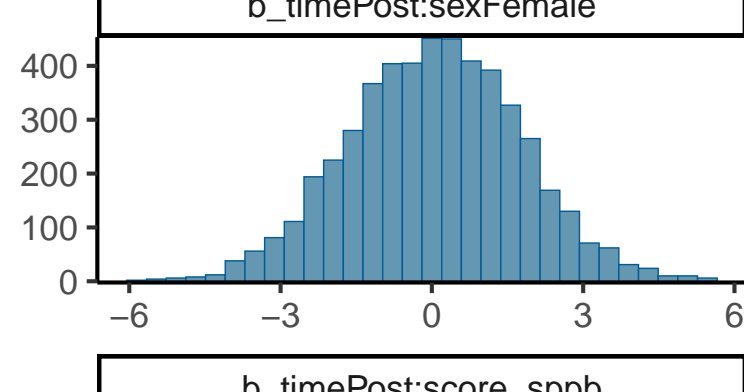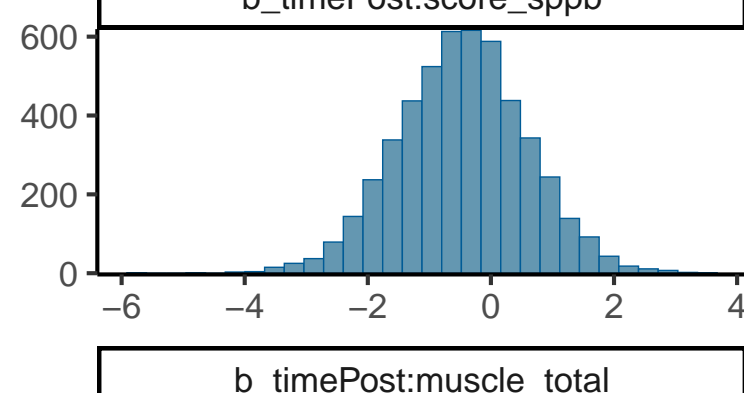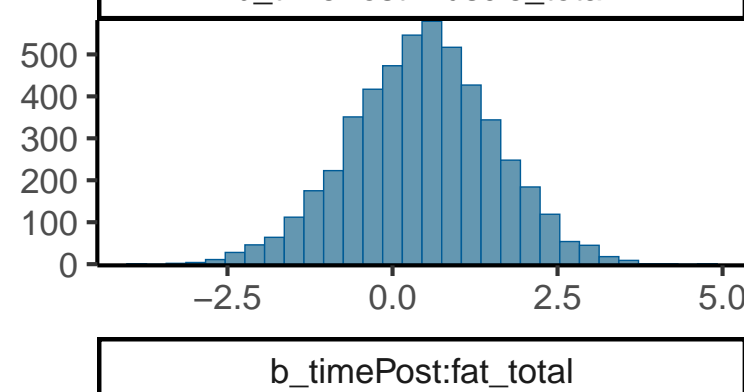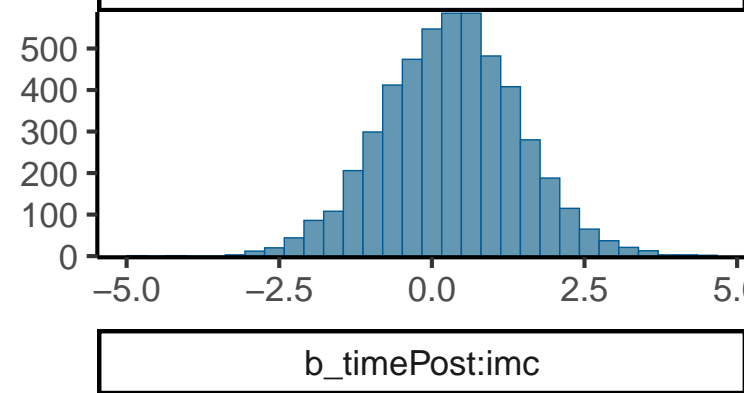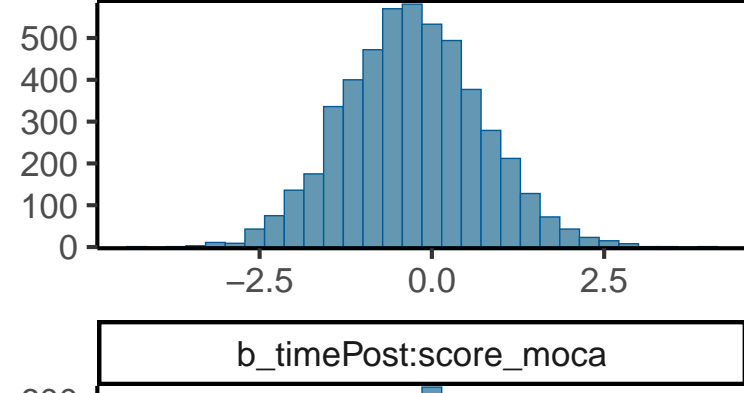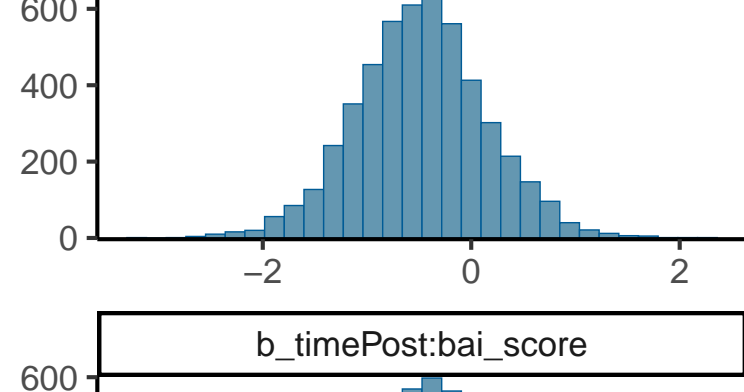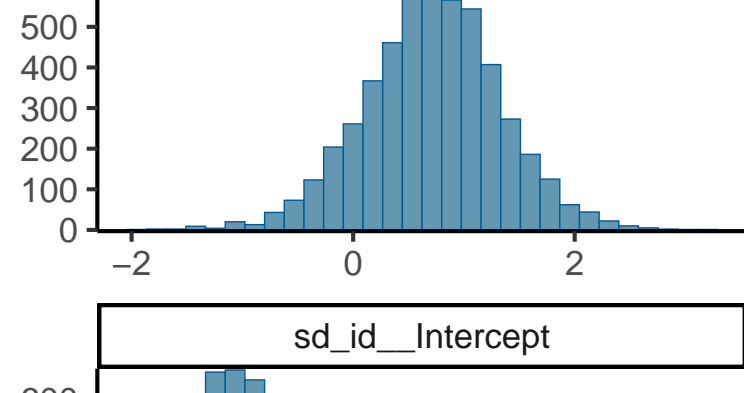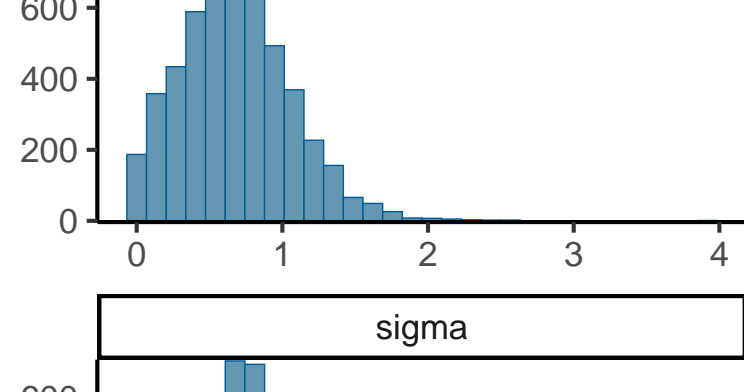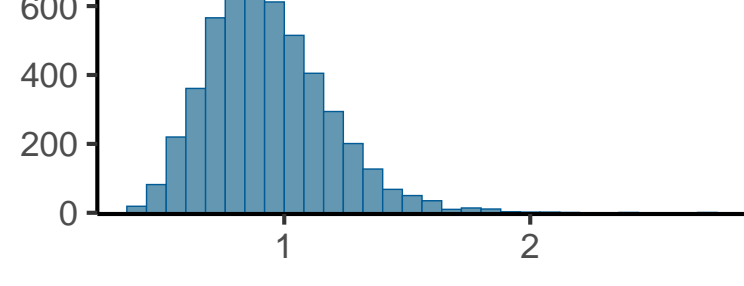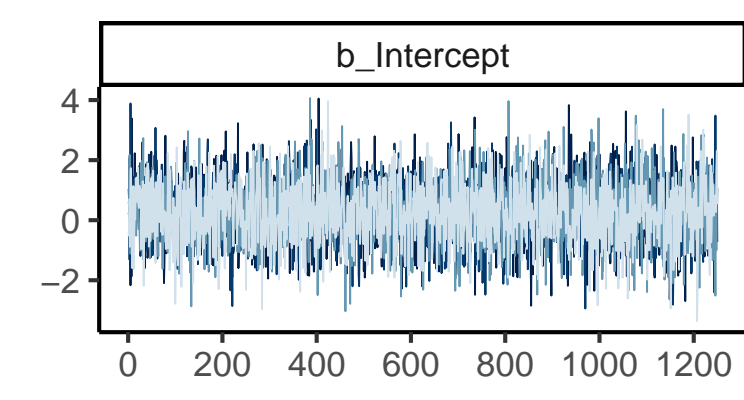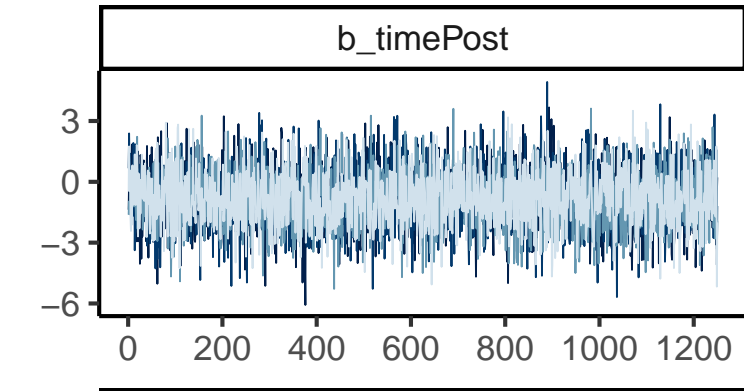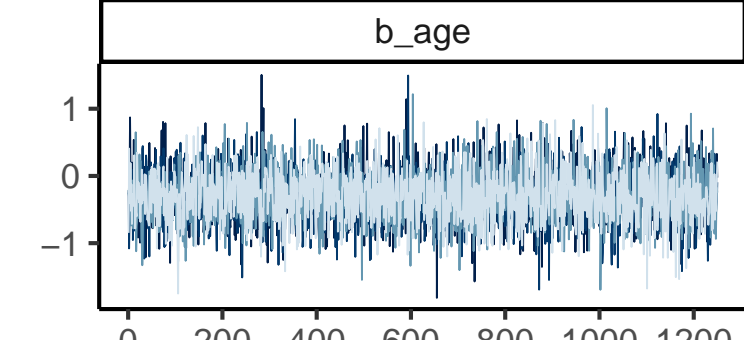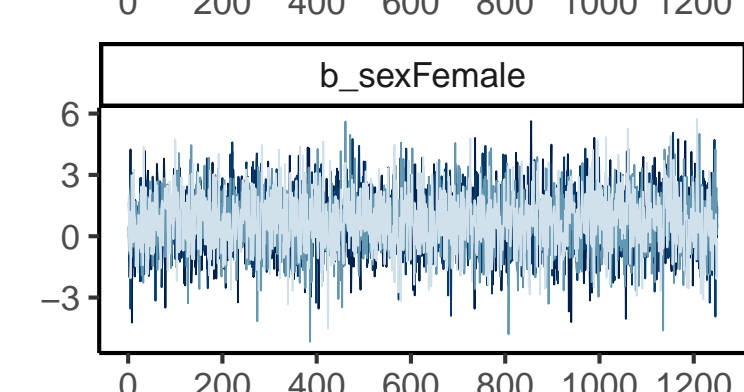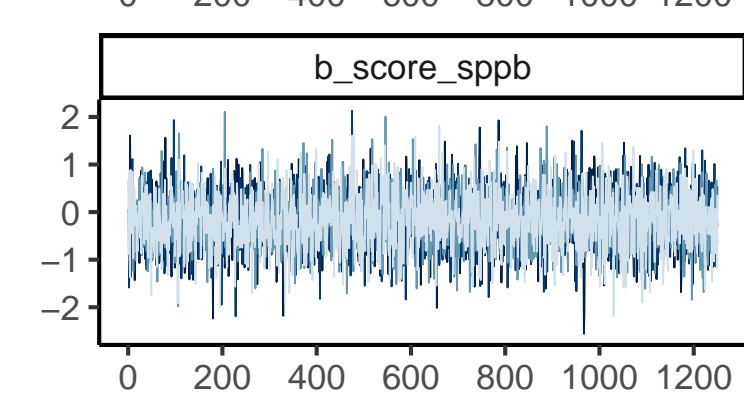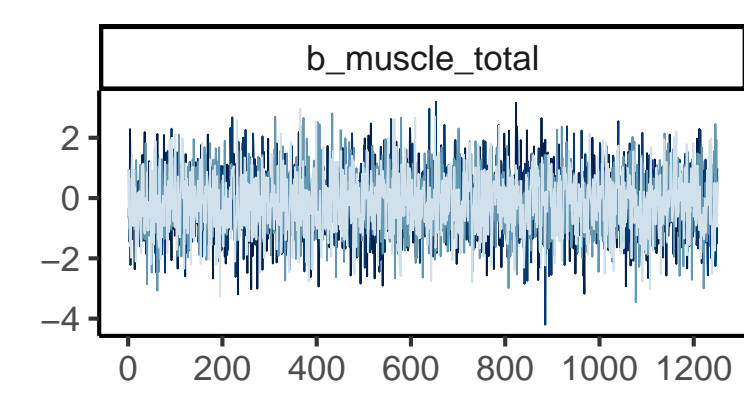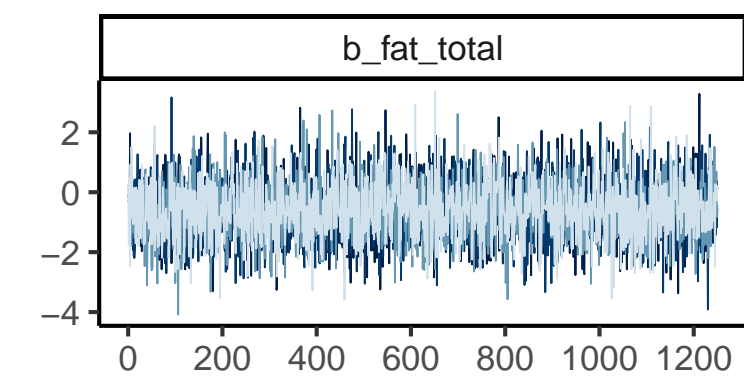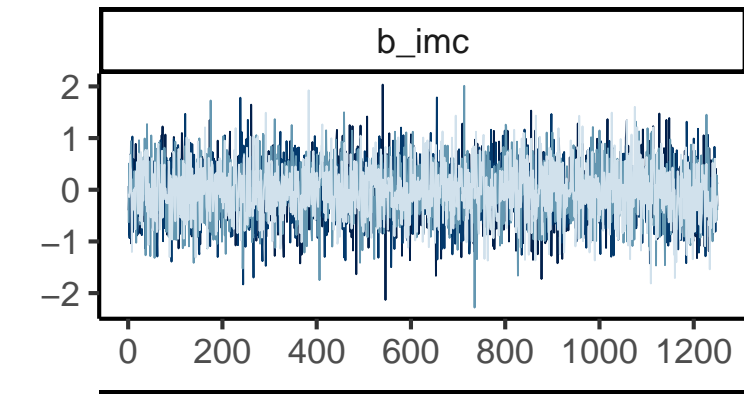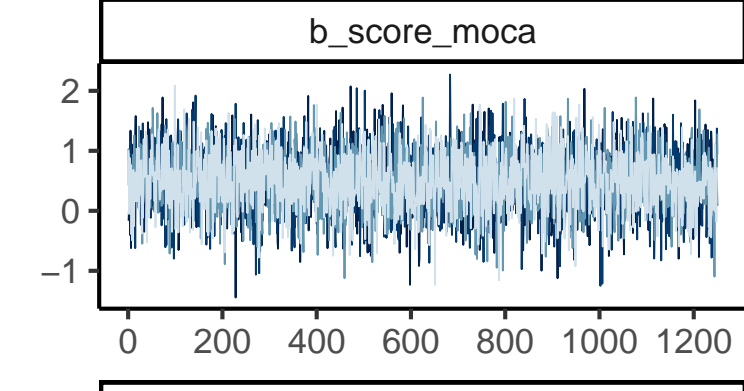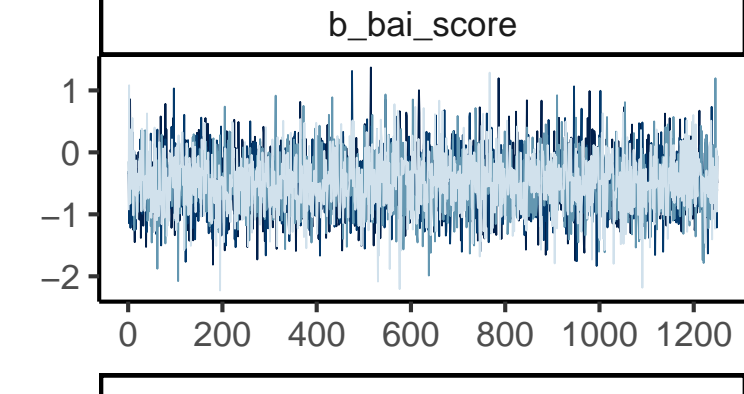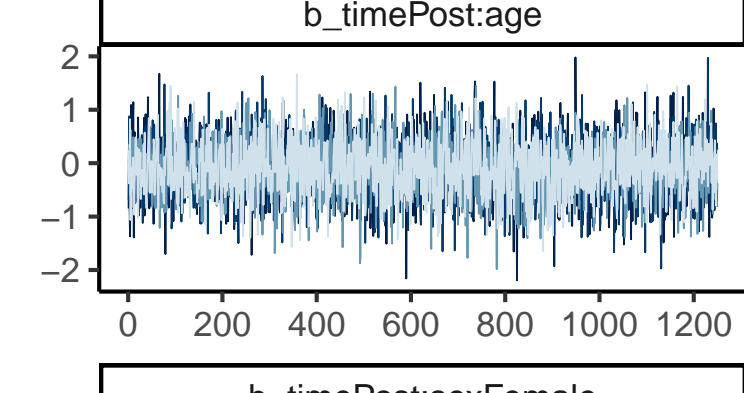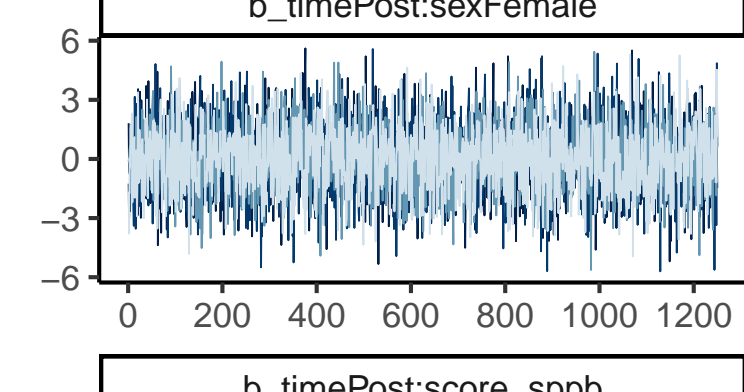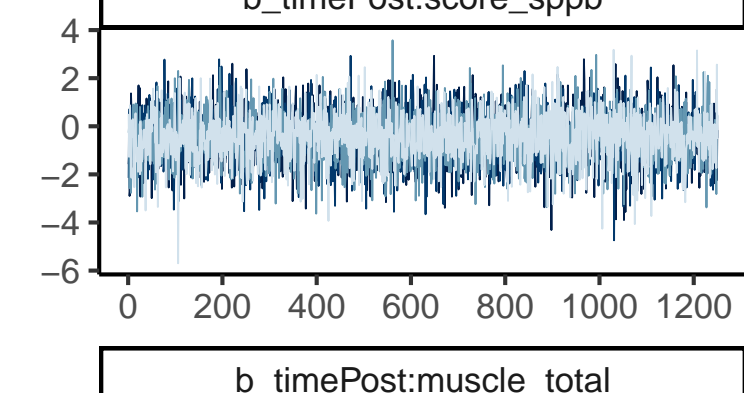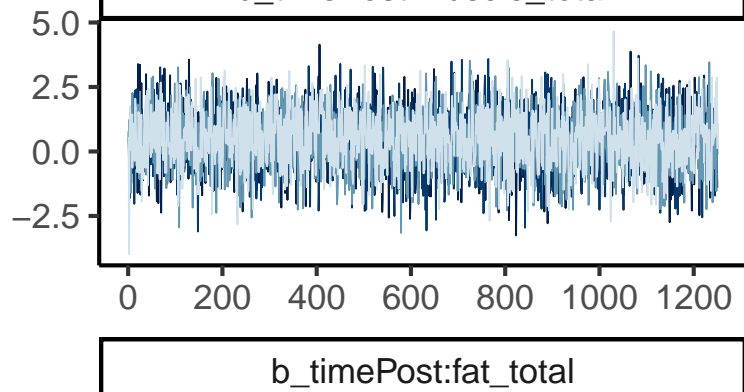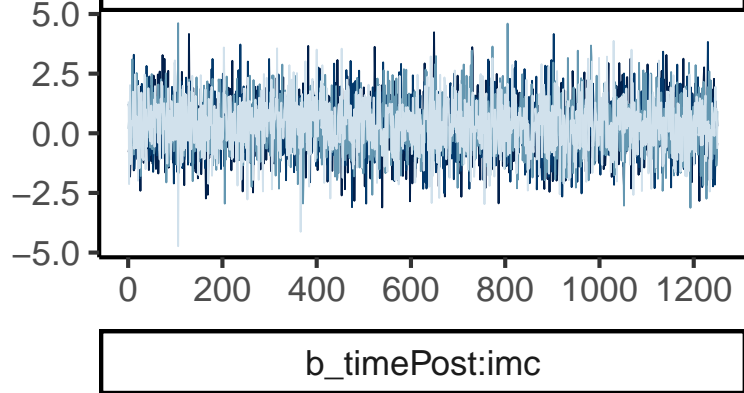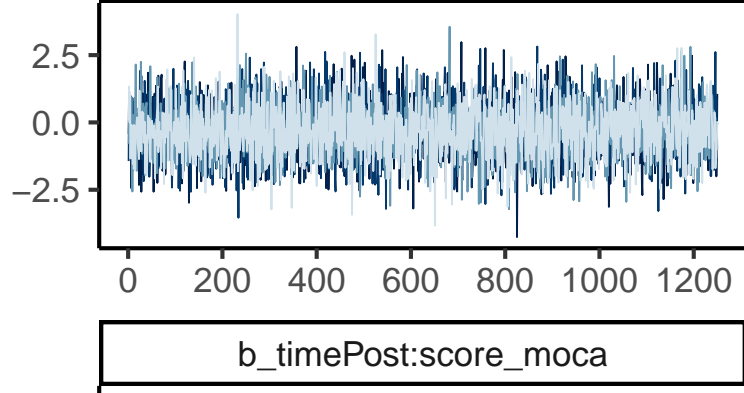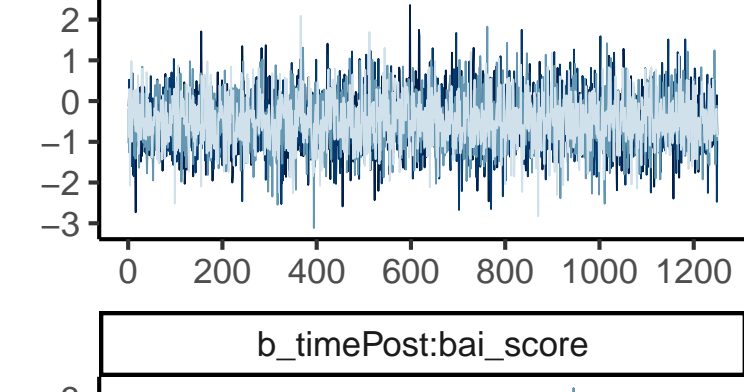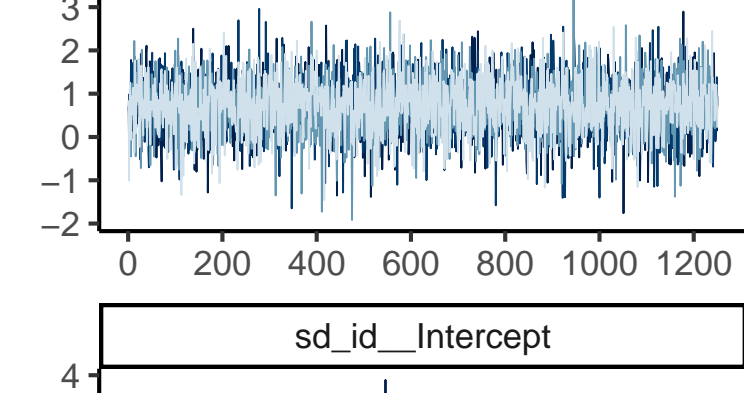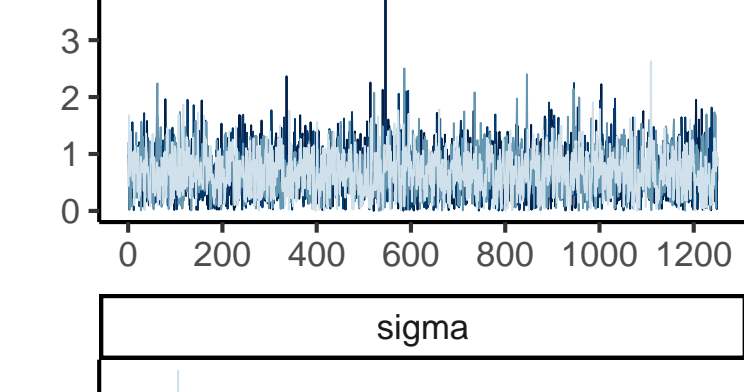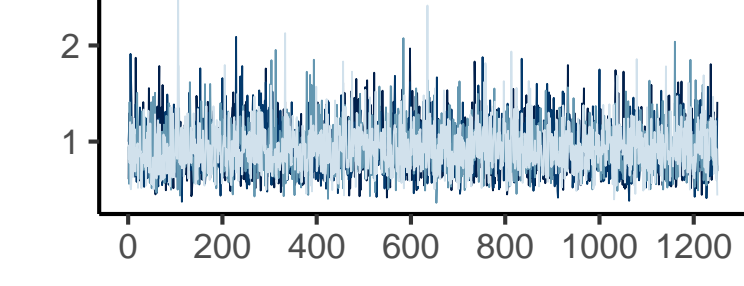

Supplement: Supplementary file 1 [file DataSheet1.pdf]

—  $y$  —  $y_{\text{rep}}$

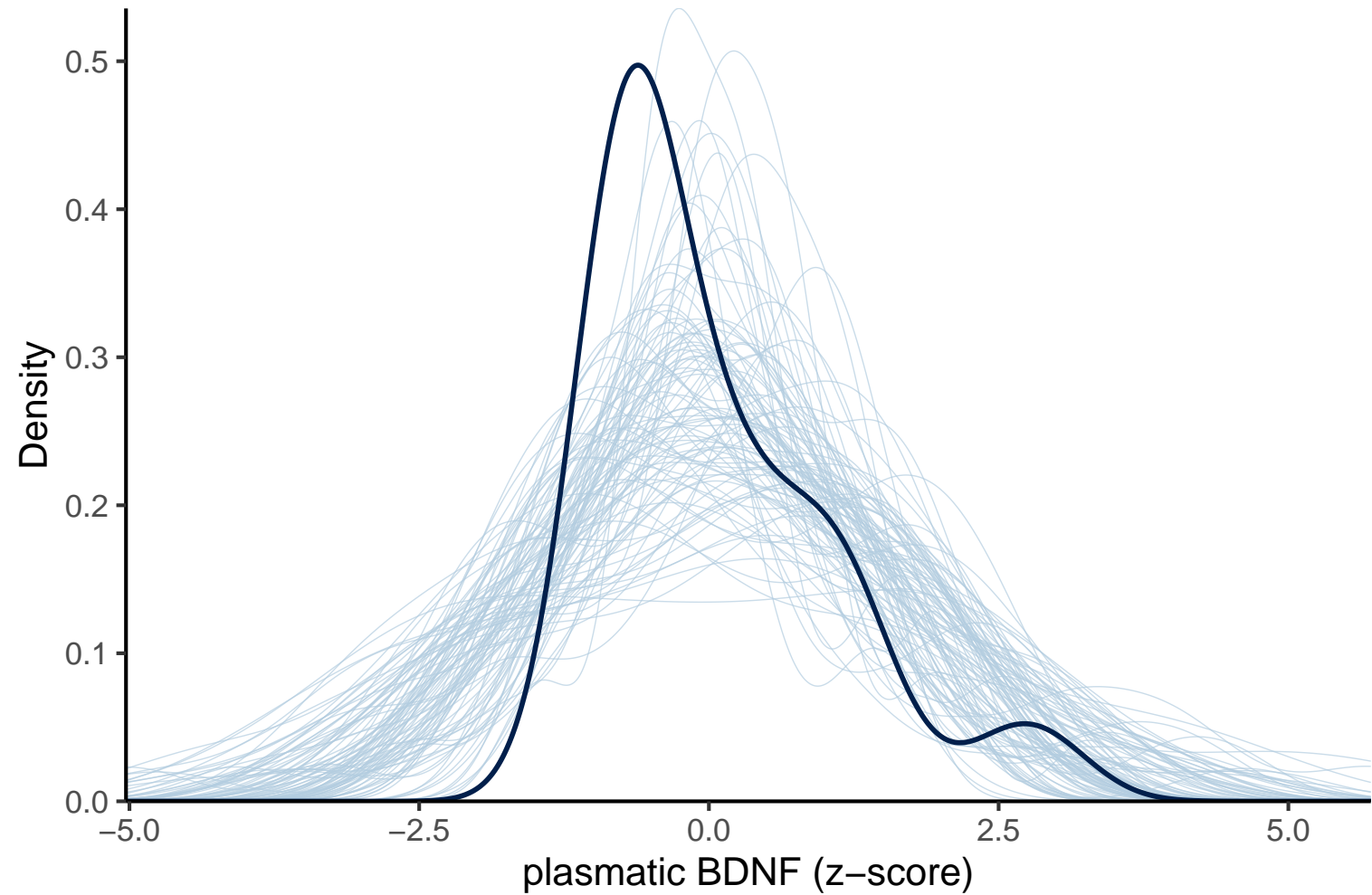

Supplement: Supplementary file 2 [file DataSheet2.pdf]
